# Construction of a cuproptosis-associated lncRNA prognostic signature for bladder cancer and experimental validation of cuproptosis-related lncRNA UBE2Q1-AS1

**DOI:** 10.3389/fmed.2023.1222543

**Published:** 2023-08-08

**Authors:** Junlin Shen, Mingyang Du, Shuang Liang, Linhui Wang, Jianbin Bi

**Affiliations:** ^1^Department of Urology, The First Hospital of China Medical University, Shenyang, Liaoning, China; ^2^Department of Radiology, Shengjing Hospital of China Medical University, Shenyang, Liaoning, China; ^3^Pharmacy Department, Hebei Medical University Third Hospital, Shijiazhuang, China

**Keywords:** cuproptosis, immune microenvironment, lncRNA, bladder cancer, risk signature, UBE2Q1-AS1

## Abstract

**Introduction:**

Bladder cancer (BLCA) is the ninth most common malignancy worldwide and the fourth most common cancer in men. Copper levels are significantly altered in patients with thyroid, breast, lung, cervical, ovarian, pancreatic, oral, gastric, bladder, and prostate cancers. Outcomes can be predicted by constructing signatures using lncRNA-related genes associated with outcomes.

**Methods:**

We identified lncRNAs related to outcomes, those differentially expressed in bladder cancer, and cuproptosis-related lncRNAs from TCGA. We identified the intersection to obtain 12 genes and established a prognostic risk signature consisting of eight genes using LASSO-penalized multivariate Cox analysis. We constructed a training set, performed survival analysis on the high-and low-risk groups, and performed validation in the test and full sets. There existed a substantial contrast in the likelihood of survival among the cohorts of high and low risk. An in-depth analysis of the gene mutations associated with tumors was conducted to evaluate the risk of developing cancer. We also performed gene analysis on neoadjuvant chemotherapy. We conducted experimental validation on the key gene UBE2Q1-AS1 in our prognostic signature.

**Results:**

The risk signature we constructed shows significant differences between the high-risk group and the low-risk group. Univariate survival analysis of the eight genes in our signature showed that each gene distinguished between high- and low-risk groups. Sub-group analysis revealed that our risk score differed significantly in tumor stage, age, and gender. The analysis results of the tumor mutation burden (TMB) showed a significant difference in the TMB between the low- and high-risk groups, which had a direct impact on the outcomes. These findings highlight the importance of TMB as a potential prognostic marker in cancer detection and prevention. We analyzed the immune microenvironment and found significant differences in immune function, validation responses, immunotherapy-related positive markers, and critical steps in the tumor immunity cycle between the high- and low-risk groups. We found that the effect of anti-CTLA4 and PD-1 was higher in the high-risk group than in the low-risk group.Gene analysis of neoadjuvant chemotherapy revealed that the treatment effect in the high-risk group was better than in the low-risk group. The key gene UBE2Q1-AS1 in our prognostic signature can significantly influence the cell viability, migration, and proliferation of cancer cells.

**Discussion:**

We established a signature consisting of eight genes constructed from cuproptosis-related lncRNAs that have potential clinical applications for outcomes prediction, diagnosis, and treatment.

## 1. Introduction

Bladder cancer (BLCA) is the ninth most common malignancy worldwide and the fourth most common cancer in men (1). As the most prevalent urinary system tumor, its incidence continues to rise in China (2). More than 90 percent of BLCAs are of the urothelial tumor type and are classified as superficial nonmuscle-invasive BLCA and muscular nonmuscle-invasive BLCA according to their clinical characteristics (3). Current treatments include radical cystectomy with extensive pelvic lymphadenectomy, immune checkpoint block immunotherapy, neoadjuvant chemotherapy, and radiotherapy. These methods are somewhat effective; however, outcomes in advanced metastatic tumors are poor (4–7). We hope to obtain new tools to predict outcomes, achieve early diagnosis, and identify sensitive treatment options. Tsvetkov and colleagues discovered that intracellular copper (Cu) induces regulated cell death distinct from those mediated by oxidative stress and called it ‘cuproptosis’ (8). Cu is a cofactor for the catalytic function of enzymes and the maintenance of structural stability; it plays a substantial role in physiological processes such as metabolism, signal transduction, and substance transport. Cu imbalances are associated with genetic diseases related to Cu metabolism and neurodegenerative diseases (9). Cu levels are significantly altered in patients with thyroid, breast, lung, cervical, ovarian, pancreatic, oral, gastric, bladder, and prostate cancers (10). Moderate intracellular Cu concentrations are toxic under certain circumstances, leading to cell death (11). Cu binds to the fatty lipoylated component of the tricarboxylic acid cycle, through which proteins bound to fatty acylation accumulate. Iron–sulfur cluster proteins are lost in a subsequent step, a mechanism that leads to proteotoxic stress and, ultimately, cell death (12). We wished to explore the role of cuproptosis in BLCA progression. Noncoding RNA molecules include long non-coding RNAs (lncRNAs), circRNAs, and microRNAs (miRs) (13). Over 15,000 long non-coding RNA genes are encoded in the human genome (14). LncRNAs are the portion of nonprotein-coding RNA transcripts longer than 200 nt. They participate in genetic regulation and are involved in the regulation of several pathological and physiological processes (15). According to the relative relationship between genes of lncRNAs and protein-coding genes in physical space, we classify them as sense lncRNAs, antisense lncRNAs, long intergenic ncRNAs, intronic lncRNAs, and bidirectional lncRNAs (16). There are two primary mechanisms by which lncRNAs function: (1) lncRNAs interact with promoters and gene regulatory regions to regulate chromatin transcription, and (2) lncRNAs sequester other regulators (e.g., the ceRNA mechanism) (17). LncRNAs are involved in cancer immune regulation and modulate the tumor microenvironment (18). Several lines of evidence suggest that lncRNAs participate in tumor progression, including BLCA. The most common manifestation is abnormal lncRNA expression. At present, computational biology and high-throughput sequencing data have been widely used in the field of biomedicine (19). We hope to use the phenomenon as a clue to construct a risk evaluation system for the outcomes, diagnosis, and treatment of BLCA.

## 2. Materials and methods

### 2.1. Gene expression and clinical data

Our study utilized data from TCGA^1^ and utilized the ‘survival,’ ‘DEseq2’ and ‘limma’ R software packages then identify 2418 BLCA outcomes-related lncRNAs (*p* < 0.05), 752 differentially expressed lncRNAs (|logFC| > 2, fdr < 0.05) and 1492 cuproptosis-related lncRNAs (cor > 0.4, *p* < 0.001). We collected gene expression data from 428 TCGA-BLCA samples and clinical data of 413 patients from TCGA-BLCA. We normalized the differentially expressed gene data using log2 (exp + 1). These rigorous data analysis methods allowed us to obtain accurate and reliable results. The lists of outcomes-related lncRNAs, differentially expressed lncRNAs, and cuproptosis-related lncRNAs intersected and then we obtained 12 genes, which were used for subsequent analysis.

### 2.2. Construction of a prognostic signature for cuproptosis-related lncRNAs

Using the expression of 12 genes, we employed Least absolute shrinkage and selection operator (LASSO) regression analysis to develop the cuproptosis-related lncRNA signature. We constructed the risk score using the following formula: (Coefficient lncRNA1 × expression of lncRNA1) + (Coefficient lncRNA2 × expression of lncRNA2) + ν + (Coefficient lncRNAn × expression lncRNAn), and applied it to each patient. Based on the best cut-off value of the risk score (0.036391179), we classified patients into high- and low-risk groups. To ensure robust and reliable results, we utilized strict and validated methods in our data analysis approach.

### 2.3. Validation of the risk score signature

We utilized Sangerbox^2^ to create risk charts and Kaplan–Meier curves. Additionally, we performed receiver operating characteristic (ROC) curve analysis to compare the conditional survival rate between groups using model classification. Through these analyses, we were able to verify the validity and reliability of our risk signature. Our approach provided a comprehensive evaluation of the prognostic value of cuproptosis-related lncRNAs in BLCA, offering important insights into the clinical applications of this molecular signature.

### 2.4. Correlation analysis between tumor mutation load and risk score signature

We downloaded tumor mutation burden (TMB)-related data from TCGA and used the R package ‘TMBcor’ to analyze the correlation of various TMB data with risk scores.

### 2.5. Immune microenvironment analysis

We used the ESTIMATE algorithm to calculate stromal and immune scores based on gene expression data, providing insights into the tumor microenvironment. The correlation analysis of staging and inflammatory factors with clustering was performed using the ‘heat map’ R package. We also tested the relationship between our risk score and immune checkpoints. The correlation between risk score and immune cells, immune process, and critical steps of the cancer immunity cycle were identified using the ‘pheatmap’ and ‘riskImmCor’ R packages.

### 2.6. Sensitivity analysis of the therapeutic target

Sensitive immunotherapeutic and neoadjuvant chemotherapy targets were identified based on the gene expression matrix using the R package ‘pRRophetic.’ The R package ‘ggpubr’ was used to draw boxplots of sensitive targets to compare the correlations with risk scores. We compared the risk scores of RB1, ARID1A, ERBB2, ATM, ERCC2, and FANCC between high- and low-risk groups and the mutation burden of these factors that participate in the neoadjuvant chemotherapy process. We also compared the associations of BLCA-associated drug target genes identified from the Drugbank database with our constructed risk signature for targeted and radiation therapies. We analyzed immunotherapy, ERBB therapy, chemotherapy, and anti-angiogenic therapy effects using the Drugbank database.

### 2.7. Culture and transfection of the UM-UC3 cell line

The UM-UC3 cell line was purchased from the Cell Bank of the Chinese Academy of Sciences (China). Cells were cultured in DMEM (high glucose) medium (Hyclone) containing 10% fetal bovine serum (FBS) at 37°C and 5% CO_2_. UBE2Q1-AS1-targeted small interfering RNA (siRNA) was purchased from JTSBIO Co. (China) to transfect the cells. The UBE2Q1-AS1 siRNA sequence was as follows:

GGAGAAACCUGAAUCAUUACAUCTCGAGAUGUAAUGAUUCAGGUUUCUCCUU.

### 2.8. Quantitative real-time PCR

The cells were subjected to RNA extraction using RNAiso Plus (Takara Biotechnology, Dalian, China) for the isolation of total RNA. Subsequently, the reverse transcription of total RNA was conducted employing the Prime Script RT Master Mix (Takara, Dalian). The resulting cRNA was subjected to qRT-PCR using the SYBR ^®^ Premix Ex TaqTM kit (Takara, Dalian). The results were analyzed on a Thermal Cycler Dice ^™^ Real-Time TP800 System (Takara, Kyoto), and GAPDH was selected as the internal reference gene. The relative gene expression level was obtained using the ΔΔCT method.

The primer sequences were as follows: UBE2Q1-AS1 (Forward primer: TCCTCTCCTCGCTACAAATGC; Reverse primer: GCTGGAAGCTCTTGCAGTCA).

### 2.9. CCK-8 assay

96-well plates were utilized for seeding UM-UC3 cells, and the Cell Technology Kit-8 (CCK-8) assay reagent (Dojindo Molecular Technologies) was added as per the prescribed guidelines of the manufacturer. Subsequently, an absorbance plate reader (Bio-Rad) was employed to measure the absorbance at a wavelength of 450 nm.

### 2.10. Cell migration assay

Transwell chambers with 8-μm pores were inserted into 24-well plates (Corning Costar, Corning, NY, United States). Each well received 600 μL of DMEM medium containing 10% FBS, while each chamber was loaded with 200 μL of FBS-free DMEM medium containing 10,000 suspended cells. Following incubation for 48 h at 37°C in 5% CO2, the inserts were washed with phosphate-buffered saline (PBS), and the membrane undersides were stained with crystal violet for 15 additional minutes. Cell passage through the membrane was measured using ImageJ installed on an inverted microscope (EVOS XL system, AMEX1200; Life Technologies Corp, Bothell, WA, United States) and images were captured at 10 × magnification.

### 2.11. Wound-healing assay

We performed a wound-healing assay in six-well plates. When UM-UC3 cells grew to a confluence higher than 90%, we scratched a straight artificial wound in the plate using a 200-μL pipette tip and incubated it with an FBS-free medium. After 24 h, cell migration was observed and photographed under a 10x lens using a microscope as previously described. Migration rate statistics were performed using ImageJ.

### 2.12. Ethynyl-20-deoxyuridine (EdU) assay

Cell proliferation was evaluated using an EdU assay kit (Ribobio, Guangzhou, China). In brief, 2000 transfected cells were seeded into 96-well plates. After 18 h, 500 μL of medium containing 50 μM EdU was added to each well, followed by incubation for 3 h. The medium was then removed, and the cells were fixed with 4% paraformaldehyde at room temperature for 15 min. Next, 0.3% Triton X-100 in PBS was added to each well to permeabilize the cells for 15 min, and a click reaction solution was added for an additional 30 min. After staining with Hoechst 33342 diluted in PBS (1:1000) for 10 min, the proportion of EdU-positive cells was determined using a fluorescence microscope (Olympus Corporation, Japan), and images were captured for subsequent analyses with ImageJ software (NIH Image, Bethesda, MD).

## 3. Results

### 3.1. Cuproptosis-related risk signature construction in BLCA

**Figure 1:**
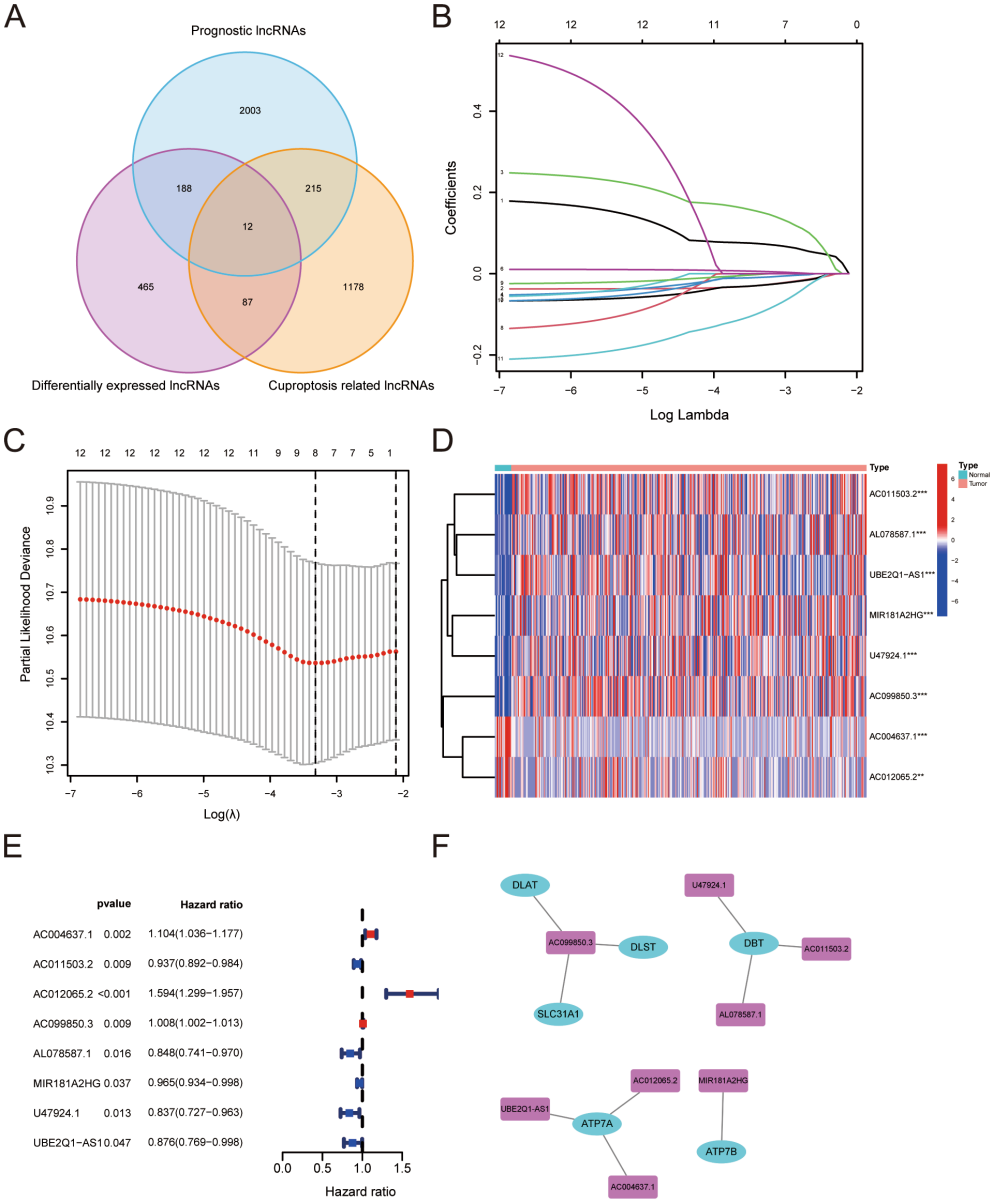
**(A)** Number and relationship of prognostic lncRNAs, differentially expressed lncRNAs and cuproptosis-related lncRNAs. **(B)** Least absolute shrinkage and selection operator coefficient spectra of 12 BLCA prognostic genes. **(C)** Optimal lambda. **(D,E)** Differential expression levels of eight genes in BLCA versus normal tissues. **(F)** Relationship between the screened risk signature and critical genes for cuproptosis.

We found 2418 lncRNAs associated with BLCA outcomes, 752 differentially expressed lncRNAs, and 1492 lncRNAs associated with cuproptosis using the R package. Twelve genes were obtained by the intersection of the three groups of lncRNAs (Figure 1A). Based on the expression levels of these 12 genes, we performed LASSO regression analysis. Ultimately, we established a prognostic signature consisting of 8 genes, according to the analysis results (Figures 1B,C). The formula was as follows:

$$\begin{array}{c} \mathrm{Risk score}=0.0731974994485369^{*}\;\mathrm{AC}004637.1\\ -\;0.0280622242754703^{*}\;\mathrm{AC}011503.2\\ +\;0.150335141799383^{*}\;\mathrm{AC}012065.2\\ +\;0.00547582364031899^{*}\;\mathrm{AC}099850.3\\ -\;0.0282712638928578^{*}\;\mathrm{AL}078587.1\\ -\;0.00103585515644202^{*}\;\mathrm{MIR}181A2\mathrm{HG}\\ -\;0.00889430248155766^{*}\;U47924.1\\ -\;0.0934708056273142^{*}\;\mathrm{UBE}2Q1-\mathrm{AS1}. \end{array}$$

The expression levels of the eight genes were significantly different between BLCA and normal tissues (Figures 1D,E). The risk signature was associated with several genes critical for cuproptosis, including DLAT, DLST, SLC31A1, DBT, ATP7A, and ATP7B (Figure 1F).

**Figure 2:**
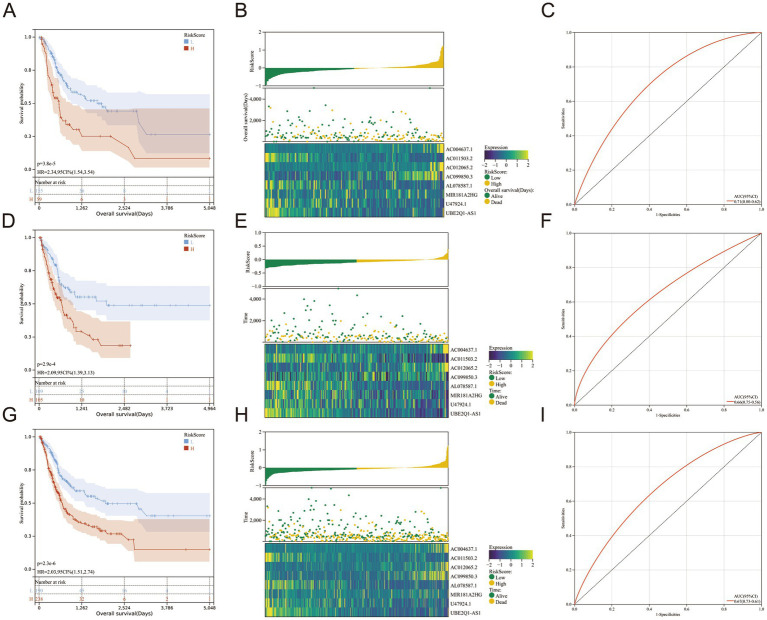
**(A–I)** The Cancer Genome Atlas training set, test set and full set were analyzed through Kaplan–Meier analysis, which revealed a significant variation in the 5-year overall survival rate between the high-risk and low-risk clusters **(A,D,G)**. The risk scores were sorted in an ascending order; patients’ over survivals; Heatmaps for eight genes in The Cancer Genome Atlas training set, test set and full set **(B,E,H)**. Receiver operating characteristic curves for 1-year overall survival in The Cancer Genome Atlas training set, test set and full set **(C,F,I)**.

We obtained data from TCGA and used them to build a training set to calculate the risk score. All BLCA patients were randomly allocated into two sets using a complete randomization method at a 1:1 ratio through SPSS software. One set served as the training set, while the other set served as the test set. The risk score had a positive relationship with the number of deaths. The survival curve demonstrated a marked contrast in 5-year overall survival (OS) between the high-risk and low-risk cohorts, revealing significant differences (Figure 2A). A heat map demonstrated that there was a significant gap in the expression of genes used to construct the risk signature for the high- and low -risk groups (Figure 2B). The area under the ROC curve (AUC) for one-year OS was 0.71 (Figure 2C). In order to confirm the findings, we established a test set and computed the risk score for both the test and full sets, subsequently classifying the samples into high- and low-risk categories. The resulting survival curve demonstrated a significant difference in the one-year OS rates in the high- and low-risk groups (Figures 2D,G). The heat map showed a significant gap in the expression of genes used to construct the risk score in the test and full sets for high and low risks (Figures 2E,H). The AUCs were 0.66 for the test set (Figure 2F) and 0.67 for the full set (Figure 2I).

**Figure 3:**
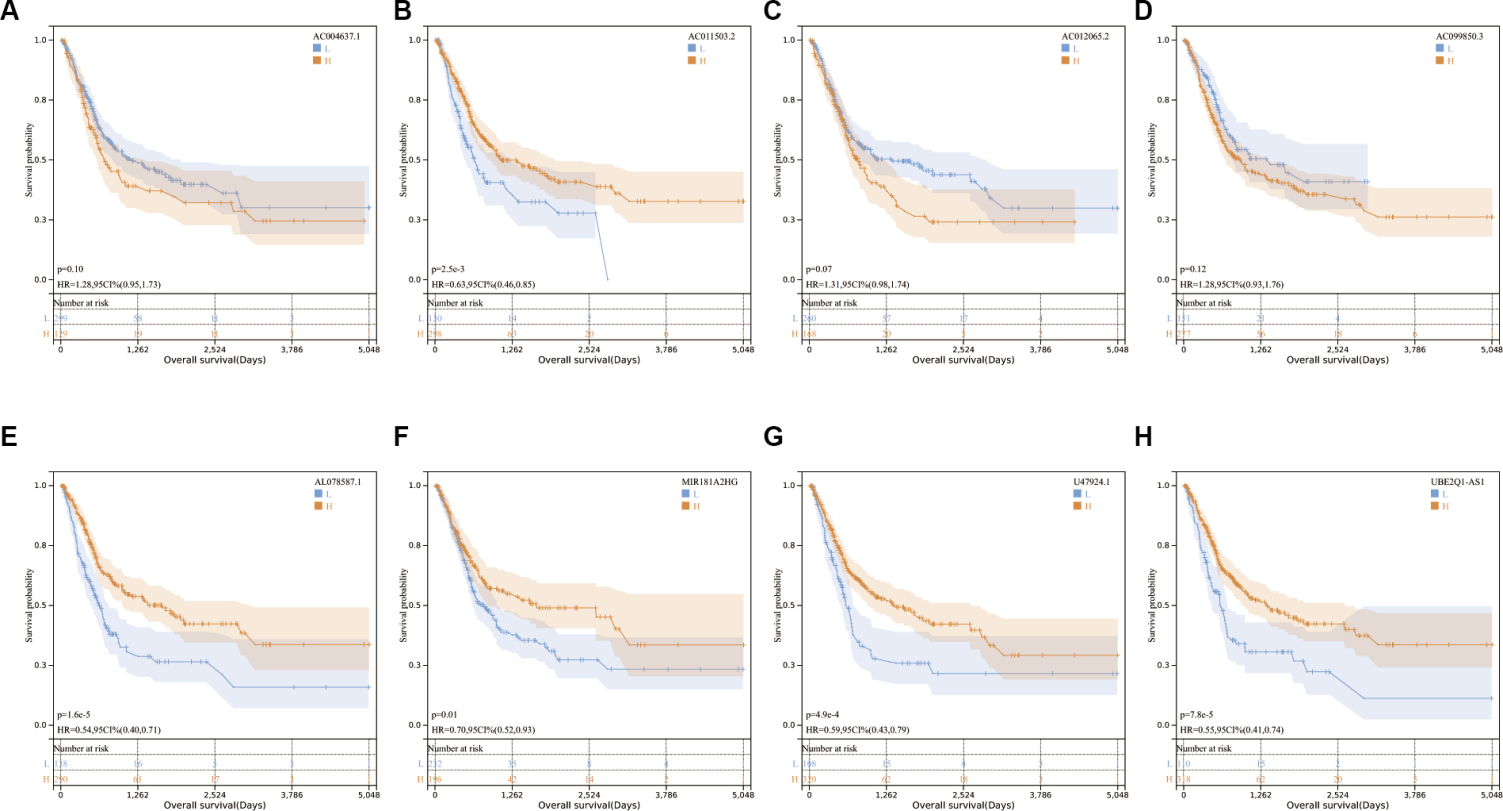
**(A–H)** Kaplan–Meier survival curves for eight genes in the full set of The Cancer Genome Atlas.

Subsequently, we conducted single-gene survival analysis for the eight genes in the full set of samples, revealing significant segregation of the high-risk and low-risk groups for each individual gene (Figures 3A–H).

Furthermore, a single-factor survival analysis was conducted on both the training and test sets, effectively discerning the high-risk group from the low-risk group in a significant manner (Supplementary Figures S1, S2).

Univariate and multivariate Cox regression analyses were conducted, revealing that age, stage, and risk score were independent risk factors in predicting outcomes (Table 1).

**Table 1:** Univariate and multivariate Cox regression analysis of age, grade, stage, and risk score.

Variables	Univariable analysis				Multivariable analysis			
	HR	95% CI of HR		*P*	HR	95% CI of HR		*P*
		Lower	Upper			Lower	Upper	
Age	1.03	1.02	1.05	4.74E-05	1.03	1.01	1.05	0.0002
Gender	0.86	0.62	1.19	0.3489	0.89	0.64	1.24	0.4983
Grade	2.89	0.72	11.69	0.1361	1.11	0.27	4.63	0.8829
Stage	1.74	1.44	2.11	1.71E-08	1.66	1.36	2.02	6.19E-07
Risk score	8.11	3.28	20.09	6.01E-06	7.71	3.04	19.56	1.71E-05

**Figure 4:**
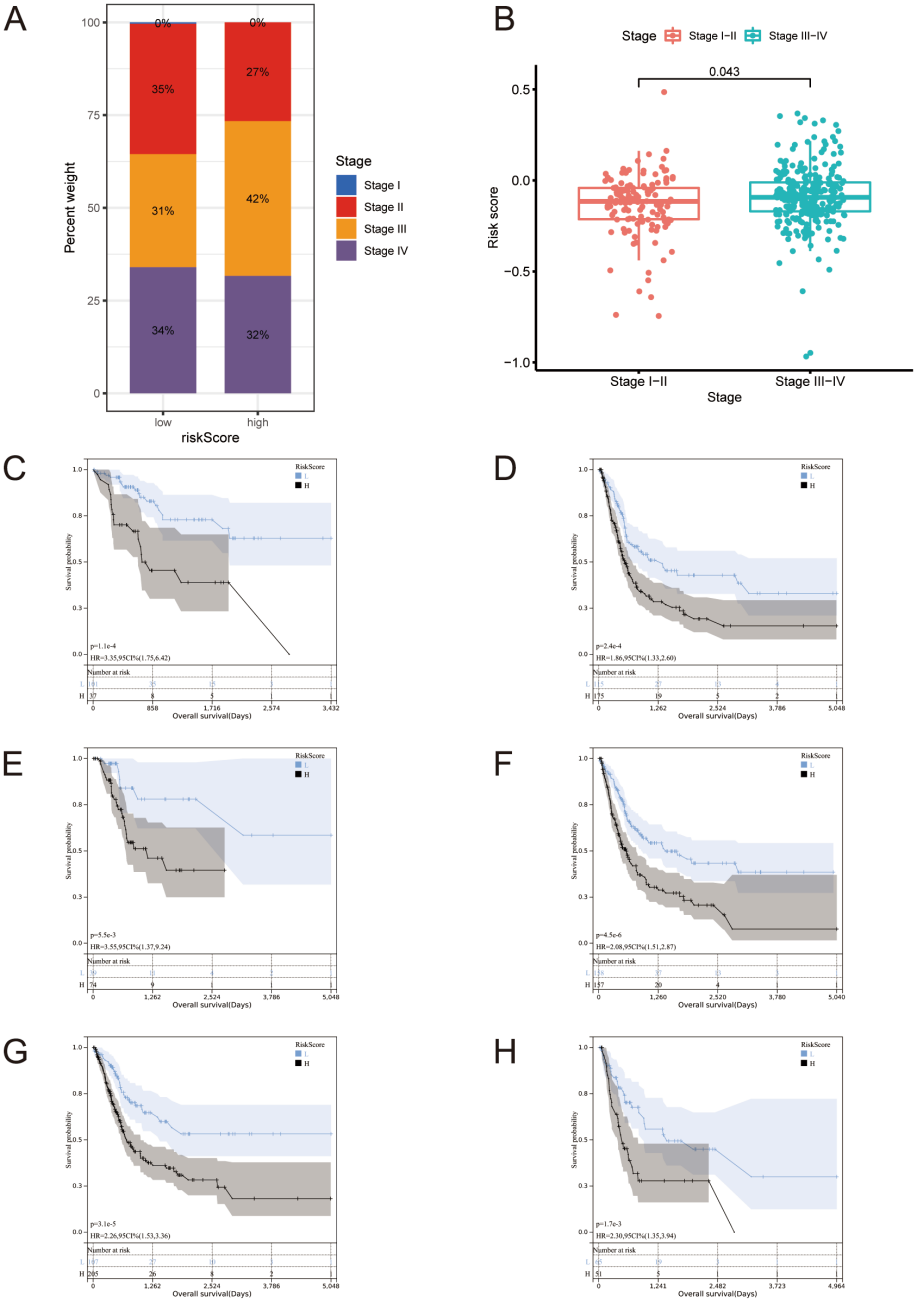
**(A,B)** Staging proportion of patients in high and low-risk groups, and risk score distribution of patients in the Stage 1 + Stage 2 + Stage 3 + Stage 4 group. It should be noted that there are samples at stage I in panel **(A)**, but due to their very small number and rounding off, they ultimately appear as 0%. **(C–H)** All BLCA cases in The Cancer Genome Atlas full set were stratified according to clinicopathological parameters. **(C)** Stage I-II; **(D)** Stage III-IV; **(E)** age ≤ 60; **(F)** age > 60; **(G)** Male; **(H)** Female.

We analyzed cancer stages in the high- and low-risk groups and analyzed the risk scores of patients in the Stage 1 + Stage 2 and Stage 3 + Stage 4 groups. There was a positive correlation between stage and risk scores (Figures 4A,B). We performed a clinical sub-groups survival analysis in the full set based on the eight genes in the genetic risk score, and compared between Stage 1 + Stage 2 and Stage 3 + Stage 4, age > 60 years and age < = 60 years, and men and women (Figures 4C–H).

These comparisons revealed significant differences between these sub-groups. We performed the same clinical sub-groups survival analysis in the training and test sets and found significant differences (Supplementary Figures S3, S4). These findings suggest that our signature is accurate and can predict outcomes.

### 3.2. Risk-based analysis of TMB

**Figure 5:**
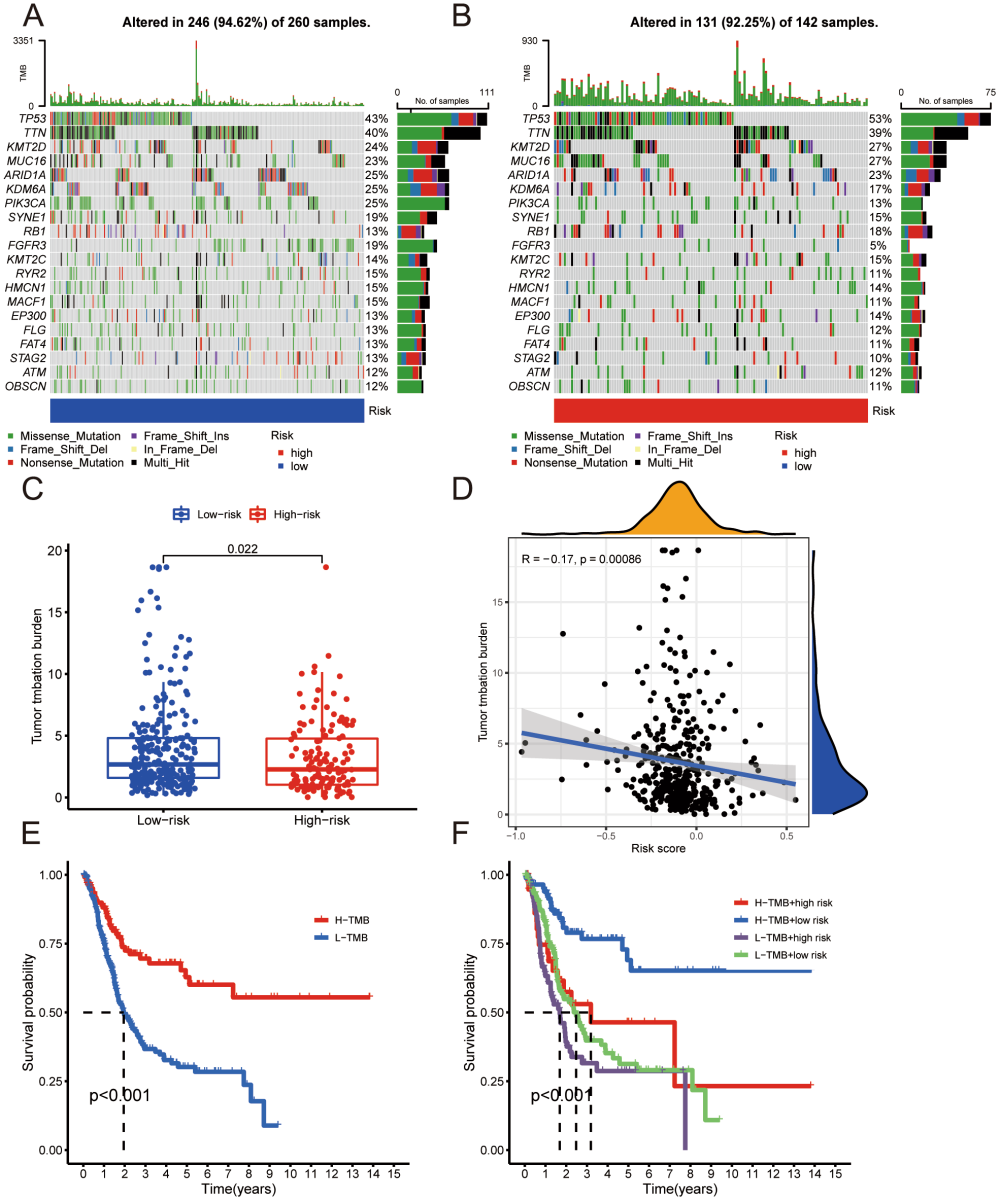
**(A)** Tumor-associated gene mutation burden in low-risk group samples. **(B)** Tumor-associated gene mutation burden in high-risk group samples. **(C)** Scatter Plot of tumor mutation burden for low- and high-risk groups. **(D)** Correlation analysis between risk score and tumor mutation burden. **(E)** Survival analysis of high tumor mutation burden group and low tumor mutation burden group. **(F)** Survival analysis of H-TMB + high-risk group, H-TMB + low-risk group, L-TMB + high-risk group, and L-TMB + low-risk group.

We analyzed the tumor-related gene mutation burden in the samples of the low- and high-risk groups and significant differences in the gene burden, gene type, and mutation (Figures 5A,B). The difference in TMB was statistically significant (Figure 5C). We performed a correlation analysis between the risk score and TMB and found that the risk score presented a significant negative relationship with TMB (Figure 5D). The survival analysis of the high- and low-TMB groups showed that the outcomes of the low-TMB group were significantly worse than that of the high-TMB group (Figure 5E). We divided the risk score and TMB as criteria for grouping (high TMB + high-risk; high TMB + low-risk; low-TMB + high-risk; low-TMB + low-risk) and performed survival analysis. The difference in survival outcomes among the groups was significant (Figure 5F), suggesting that risk score and TMB could be used as independent risk assessment methods to predict BLCA outcomes, and the risk score predicts TMB.

### 3.3. Risk-based analysis of the immune microenvironment

**Figure 6:**
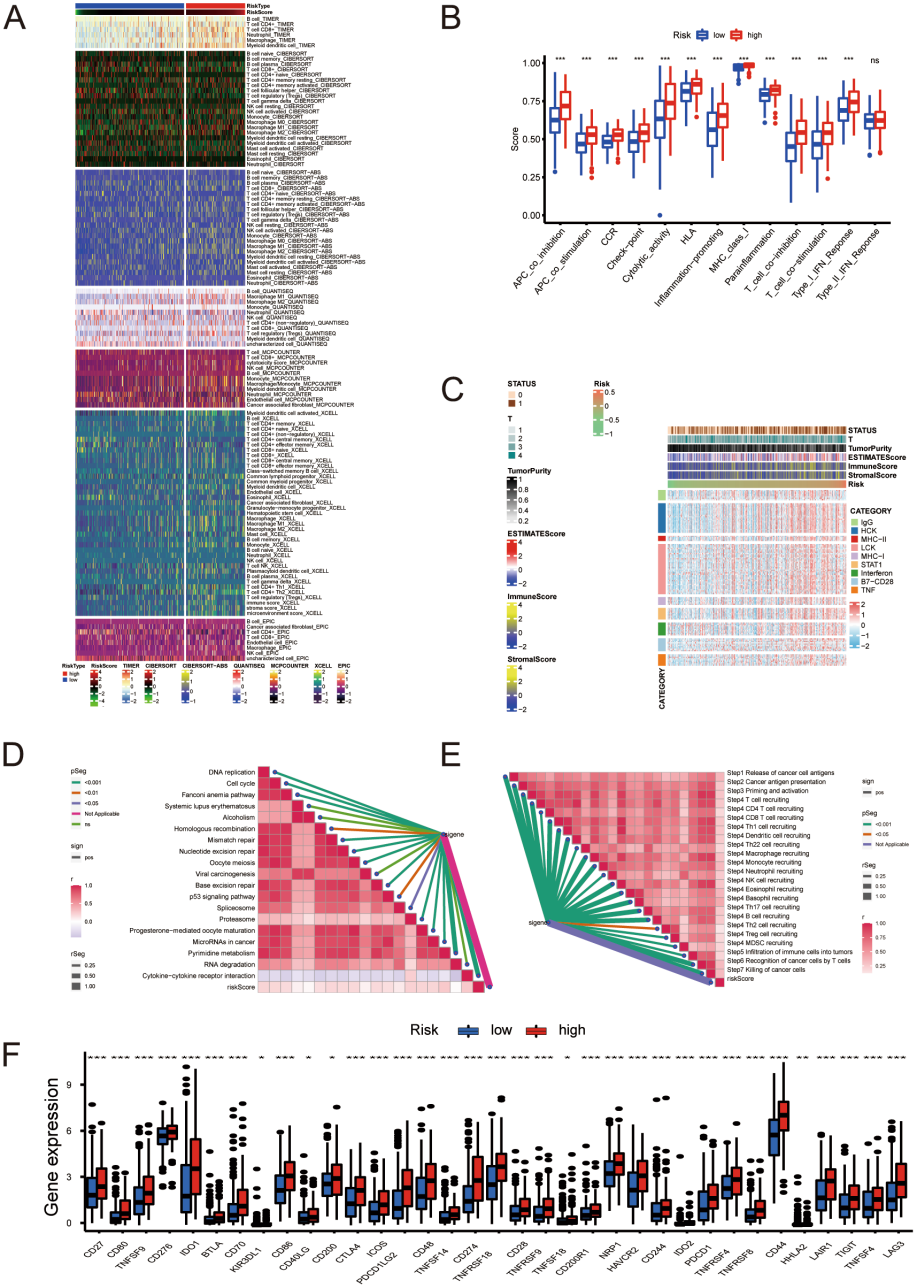
**(A)** Relationship between molecular subtypes and immune molecular typing. **(B)** Analysis of immune function in the low- and high-risk groups. **(C)** Inflammatory response analysis. **(D)** Correlation analysis between risk score and positive markers related to immunotherapy. **(E)** Analysis of the correlation between risk scores and critical steps of the cancer immunity cycle. **(F)** Immune checkpoint analysis. * Represents *P* ≤ 0.05; ** Represents *P* ≤ 0.01; *** Represents *P* ≤ 0.001.

We analyzed the immune microenvironment of samples with different risk scores and obtained the expression profiles of their immune microenvironments (Figure 6A). We then analyzed the immune function of the high- and low-risk groups. The high-risk group’s immune function was significantly more hyperactive than the low-risk group (Figure 6B). The inflammatory response is associated with immunotherapy; therefore, we performed an analysis of the inflammatory response and found that the inflammatory response in the high-risk group was more intense than that of the low-risk group (Figure 6C). The risk signature we constructed was positively correlated with the enrichment score of all immunotherapy-related positive markers (Figure 6D). The risk signature presents a positive relationship with the critical steps of the cancer immunity cycle (Figure 6E). The high- and low-risk groups were subject to immune checkpoint analysis, which uncovered higher gene expression of immune checkpoints in the former than in the latter (Figure 6F). These findings suggest the possibility that samples in the high-risk group might obtain higher sensitivity and better therapeutic effects in terms of immunotherapy.

**Figure 7:**
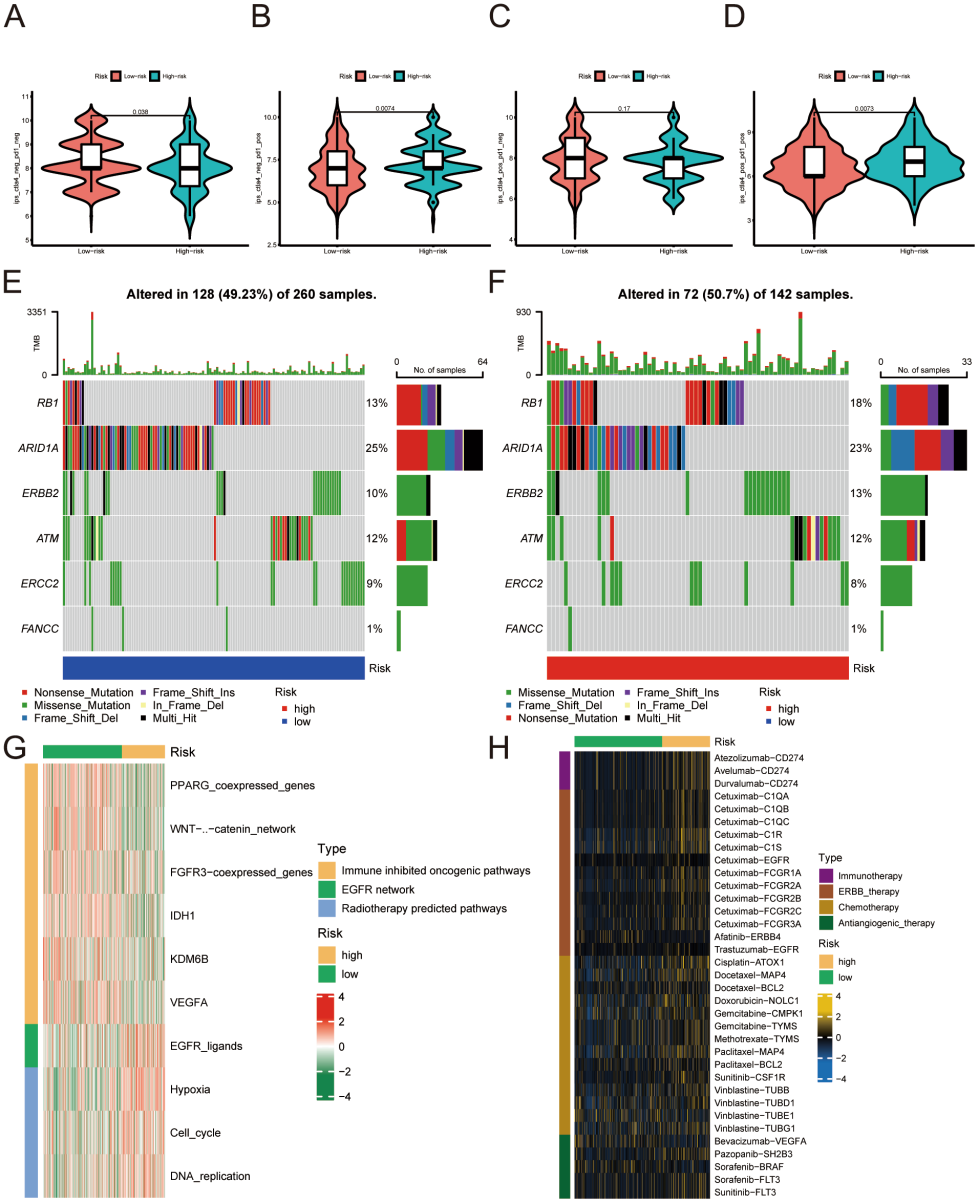
**(A–D)** No anti-CTLA4 and PD-1, anti-CTLA4 only, anti-PD-1 only, combined with anti-CTLA4 and PD-1 treatment effect scores in the low- and high-risk groups. **(E)** Expression profile of genes associated with neoadjuvant chemotherapy in the low-risk group. **(F)** Expression profile of genes related to neoadjuvant chemotherapy. **(G)** Enrichment analysis related to radiotherapy prediction pathways and targeted therapy in the low- and high-risk groups. **(H)** Enrichment analysis of samples from low- and high-risk groups for drug targets, including immunotherapy, ERBB therapy, chemotherapy, and anti-angiogenic therapy.

Based on our analysis of immune checkpoints, the high- and low-risk groups showed a significant difference, and we focused on CTLA4 and PD-1 (targets for cancer immunotherapy). We divided the samples according to treatment regimens: no anti-CTLA4 and PD-1, only anti-CTLA4, only anti-PD-1, and anti-CTLA4 and PD-1 combined. Our study compared the treatment effect scores of anti-CTLA4 or PD-1 treatment regimens between high- and low-risk groups. The results indicated that patients in the high-risk group who received single or combined anti-PD-1 checkpoint therapy showed significantly better treatment effects than those in the low-risk group. This finding suggests that the immunotherapy effect of anti-CTLA4 and PD-1 in the high-risk group was better than that of the low-risk group (Figures 7A–D). The finding also partly corroborates our previous conjecture that our risk score can predict the sensitivity and effectiveness of immunotherapy. We analyzed the expression of neoadjuvant chemotherapy-related genes in various groups according to the risk scores and found that mutations of RB1 and ERBB2 in the high-risk group were significantly more numerous than in the low-risk group (Figures 7E,F). This finding suggests that patients with higher risk scores have better neoadjuvant treatment outcomes. The enrichment score of the radiotherapy prediction and targeted therapy pathways were higher in the high-risk group than in the low-risk group (Figure 7G). The Drugbank database revealed that the samples in the high-risk group responded significantly to immunotherapy, ERBB therapy, chemotherapy, and anti-angiogenic therapy (Figure 7H). The data indicates that the therapeutic benefit for patients undergoing immunotherapy, neoadjuvant chemotherapy, radiotherapy, and anti-angiogenic therapy is significantly higher in the high-risk group compared to the low-risk group.

### 3.4. UBE2Q1-AS1 knockdown significantly inhibited BLCA cell viability, migration, and proliferation

**Figure 8:**
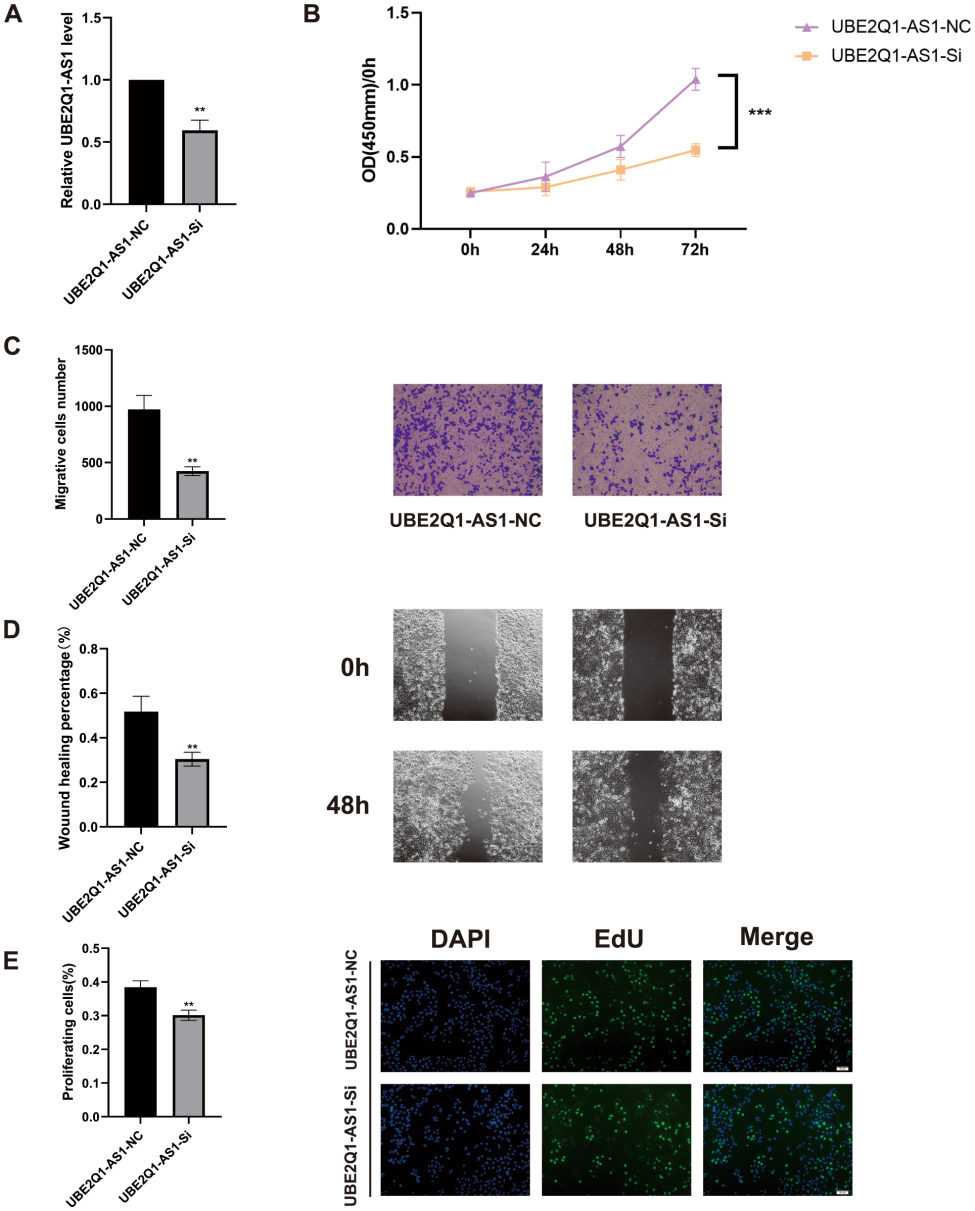
**(A)** Real-time quantitative PCR before and after UBE2Q1-AS1 knockdown. **(B)** A CCK8 assay was used to show that the knockdown of UBE2Q1-AS1 inhibited viability in UM-UC3 cells. **(C)** Cell migration assays demonstrated the effect of UBE2Q1-AS1 on UM-UC3 cells migration. **(D)** Wound-healing assay was used to test the effect of UBE2Q1-AS1 on UM-UC3 cells migration. **(E)** Knockdown of UBE2Q1-AS1 inhibited the proliferation of UM-UC3 cells using an EdU assay. ** Represents *P* ≤ 0.01; *** Represents *P* ≤ 0.001.

We first knocked down UBE2Q1-AS1 using siRNA (Figure 8A). Subsequently, CCK-8 assays were conducted on UM-UC3 cells which revealed that the UBE2Q1-AS1 knockdown group exhibited significantly lower cell viability compared to the negative control group (Figure 8B). We also performed cell migration assay and wound-healing assay and found that the migration ability of UM-UC3 cells in the UBE2Q1-AS1 knockdown group was significantly lower (Figures 8C,D). Moreover, proliferation in UM-UC3 cells was markedly suppressed upon knockdown of UBE2Q1-AS1, as evidenced by the EdU assay (Figure 8E).

## 4. Discussion

Studies on lncRNA function revealed many mechanisms, and it is currently believed that lncRNAs are inextricably related to cellular functions such as differentiation, apoptosis, and proliferation (20). As a novel regulated cell death, cuproptosis is compelling regarding prognostic signature establishment related to BLCA. Understanding the mechanism of cuproptosis, we realize that it plays a critical role in BLCA progression. LncRNAs are involved in tumor development; therefore, we used bioinformatics analysis to identify lncRNAs affecting BLCA outcomes, lncRNAs differentially expressed in BLCA, and lncRNAs associated with cuproptosis. The construction of risk signature has a wide range of application value in urinary system tumors (21). We constructed a risk evaluation system for outcomes to guide diagnosis and treatment. We obtained 12 genes in TCGA using univariate Cox analysis and a risk prediction signature composed of eight genes (AC004637.1, AC011503.2, AC012065.2, AC099850.3, AL078587.1, MIR181A2HG, U47924.1, and UBE2Q1-AS1) using LASSO-penalized multivariate Cox analysis. Downregulation of miR181A2HG downregulated AKT2 expression by decreasing its sponging of miR-8056, miR-6842-5p, and miR-6832-5p, which in turn significantly inhibited the migration, proliferation, and capillary-like structure formation in human umbilical vein endothelial cells; glucose uptake, ATP content and glycogen synthesis were also significantly reduced, suggesting that MIR181A2HG also plays a critical role in energy metabolism (22). UBE2Q1-AS1 is involved in RNA degradation and ubiquitin-mediated proteolysis. Zhang et al. suspected that UBE2Q1-AS1 might play a role in cancer through the sense gene UBE2Q1, which significantly correlates with gastric cancer tumor grade (23). By conducting survival analysis, we observed notable differences in the five-year survival rates and area under the ROC curve between the high- and low-risk groups based on our devised risk scores. Our monogenic survival analysis of the eight genes revealed that they were capable of identifying and differentiating the high- and low-risk groups, while their dysregulation emerged as a standalone risk factor for BLCA. We performed survival analysis of stage, age, and gender and found that the high- and low-risk groups significantly distinguished Stage 1 + Stage 2 from Stage 3 + Stage 4, age > 60 years from age < = 60 years, and males from females. Across all human cancers, TMB predicts benefits from immune checkpoint inhibition (24); it has been proposed as a possible immunotherapy biomarker (25). Therefore, we hoped to determine if there were TMB differences between patients in the high- and low-risk groups. We found a significant gap in TMB between groups with a statistically negative relationship. The results of the survival analysis indicated that the group with a higher TMB exhibited superior outcomes compared to the group with a lower TMB. We divided the cohort into four groups according to TMB and risk signature. Survival analysis showed that TMB and risk signature were independent methods to predict BLCA outcomes, and the risk signature predicts the degree of TMB. The tumor immune microenvironment is essential in cancer occurrence and progression, and immune dysfunction is a primary cause of tumorigenesis, suggesting that correct expression of immune genes is essential for collective immune function. Prior research has mainly centered on coding genes, however, a number of studies have highlighted the importance of lncRNAs in the immune system (26–28). Therefore, we explored the immune microenvironment based on the risk signature. Wang et al. identified biomarkers in the tumor immune microenvironment using computational biology methods, which provided methodological support for our study (29). We used seven databases to obtain immune microenvironment expression profiles and found significant differences in the immune microenvironment between the high- and low-risk groups. The high-risk group exhibited heightened immune function and inflammatory responses compared to the low-risk group. The risk scores were positively correlated with immunotherapy-related positive markers, critical steps of cancer immunity circulation, and critical gene expression levels of immune checkpoints. Immune checkpoint related therapies have great value for urinary system tumors (30). Immune checkpoints regulate the initiation and maintenance of immune responses, including a series of structures located on the cell surface with similar functions exemplified by CTLA4 and PD-1 (31). Immune checkpoint inhibitors (ICI) have been approved to treat many tumors and have become a critical link in cancer therapy. The effective combination of dual ICI therapy and the use of regimens combining targeted therapy and chemotherapy is expanding (32). CTLA4 and PD-1 are the most clinically relevant checkpoints that inhibit anticancer immune responses; inhibition of these receptors has been shown to eliminate tumors in animal models (33). Based on our immune checkpoint analysis, we will focus on the analysis of CTLA4 and PD-1. By computing the treatment effect scores of four treatment regimens associated with CTLA4 and PD-1 in both high- and low-risk groups, we determined that anti-PD-1 monotherapy and anti-CTLA4 combined with PD-1 had superior treatment effects in the high-risk group compared to the low-risk group. Neoadjuvant chemotherapy is also widely used. Cisplatin-based neoadjuvant chemotherapy is often administered before radical cystectomy in patients with muscle-invasive BLCA (34, 35). Studies have shown that mutations in ERCC2, ERBB2, and DNA repair genes can predict responses to neoadjuvant chemotherapy, which is more conducive to weighing the benefits against substantial toxicity (36). After examining the gene expression levels related to neoadjuvant chemotherapy, we observed a higher incidence of RB1 and ERBB2 mutations in the high-risk group compared to the low-risk group. Based on this finding, we hypothesized that the high-risk group would gain more benefits from neoadjuvant chemotherapy. We conducted meaningful analyses of immune-targeted therapy pathways, radiation therapy-related pathways, and relevant drug targets for multiple targeted therapies and demonstrated the validity of our risk signature. We focused on BLCA outcomes prediction using a cuproptosis-related lncRNA signature and the effect of TMB, immune microenvironment analysis, immune-targeted therapy, neoadjuvant chemotherapy, and other treatments. We are the first to analyze the prognostic impact of cuproptosis in BLCA. The treatment outcomes analysis has high feasibility for clinical decision-making. We verified the conclusions from the correlation analysis experimentally. We knocked down UBE2Q1-AS1 and validated the knockdown efficiency using PCR. UBE2Q1-AS1 knockdown in UM-UC3 cells attenuated viability, migration, and proliferation. Our study has some limitations. First, our clinical data are derived from the same database, and our training and validation sets are derived from different parts of the same data source. Second, we did not include factors that may affect outcomes, such as smoking. Third, the study was retrospective; prospective, multicenter studies are needed for support and validation. Fourth, our BLCA outcomes study of cuproptosis-related lncRNAs requires in-depth related mechanistic studies. Finally, our conclusions concerning drug therapy for BLCA need to be verified in clinical trials.

## 5. Conclusion

We analyzed the genes associated with cuproptosis-related lncRNAs and constructed an eight-genes signature for BLCA, which has value in predicting BLCA outcomes and selecting targeted therapy or neoadjuvant chemotherapy.

## Data availability statement

The original contributions presented in the study are included in the article/Supplementary material, further inquiries can be directed to the corresponding author.

## Author contributions

JS, MD, SL, LW, and JB designed the study. JS and LW analyzed and wrote the manuscript. MD provided partial code support, as well as made constructive modifications to the article. SL has made contributions to the code modifications and uploads during the editing process. All authors read and agreed to the final version of the manuscript.

## Funding

This study was supported by the National Natural Science Foundation of China (Grant No. 82172568).

## Conflict of interest

The authors declare that the research was conducted in the absence of any commercial or financial relationships that could be construed as a potential conflict of interest.

## Publisher’s note

All claims expressed in this article are solely those of the authors and do not necessarily represent those of their affiliated organizations, or those of the publisher, the editors and the reviewers. Any product that may be evaluated in this article, or claim that may be made by its manufacturer, is not guaranteed or endorsed by the publisher.

## Footnotes

^1^https://portal.gdc.cancer.gov/

^2^http://sangerbox.com/
