# Supplementary material for: Construction of a cuproptosis-associated lncRNA prognostic signature for bladder cancer and experimental validation of cuproptosis-related lncRNA UBE2Q1-AS1
Source: Front Med (Lausanne). 2023 Aug 8;10:1222543. doi: 10.3389/fmed.2023.1222543 (PMC10442536; doi:10.3389/fmed.2023.1222543)
Supplement: Supplementary file 1 [file Data_Sheet_1.PDF]

Supplementary Material

Construction of a Cuproptosis-Associated lncRNA Prognostic Signature for Bladder Cancer and experimental validation of cuproptosis-related lncRNA UBE2Q1-AS1

Junlin Shen[1], Linhui Wang[1], Jianbin Bi[1*]

[1]Department of Urology, China Medical University, The First Hospital of China Medical University, Shenyang, Liaoning, China

*** Correspondence:** Jianbin Bi: jianbinbi@cmu.edu.cn

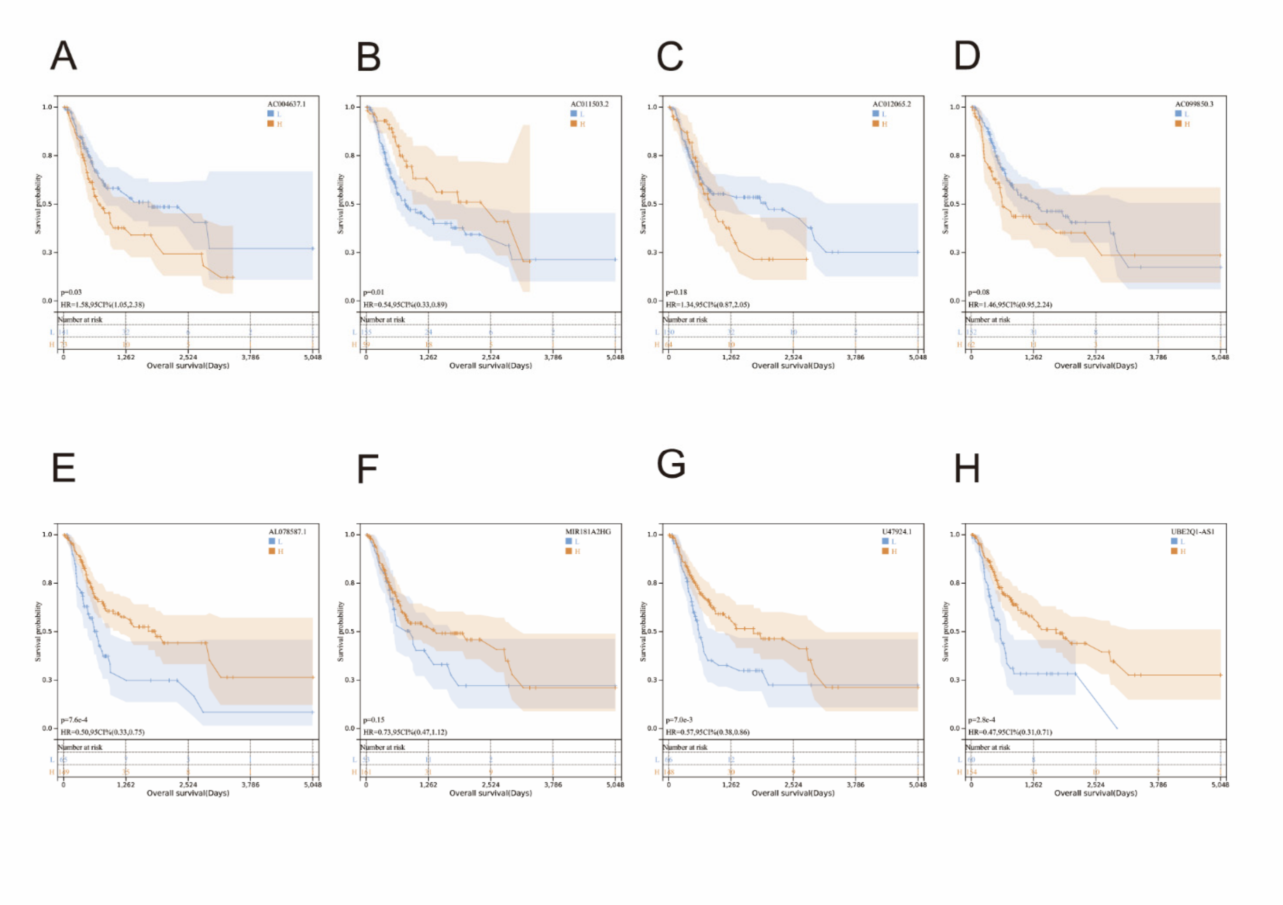

Supplementary Figure 1. A-H: Kaplan-Meier survival curves for eight genes in The Cancer Genome Atlas training set.

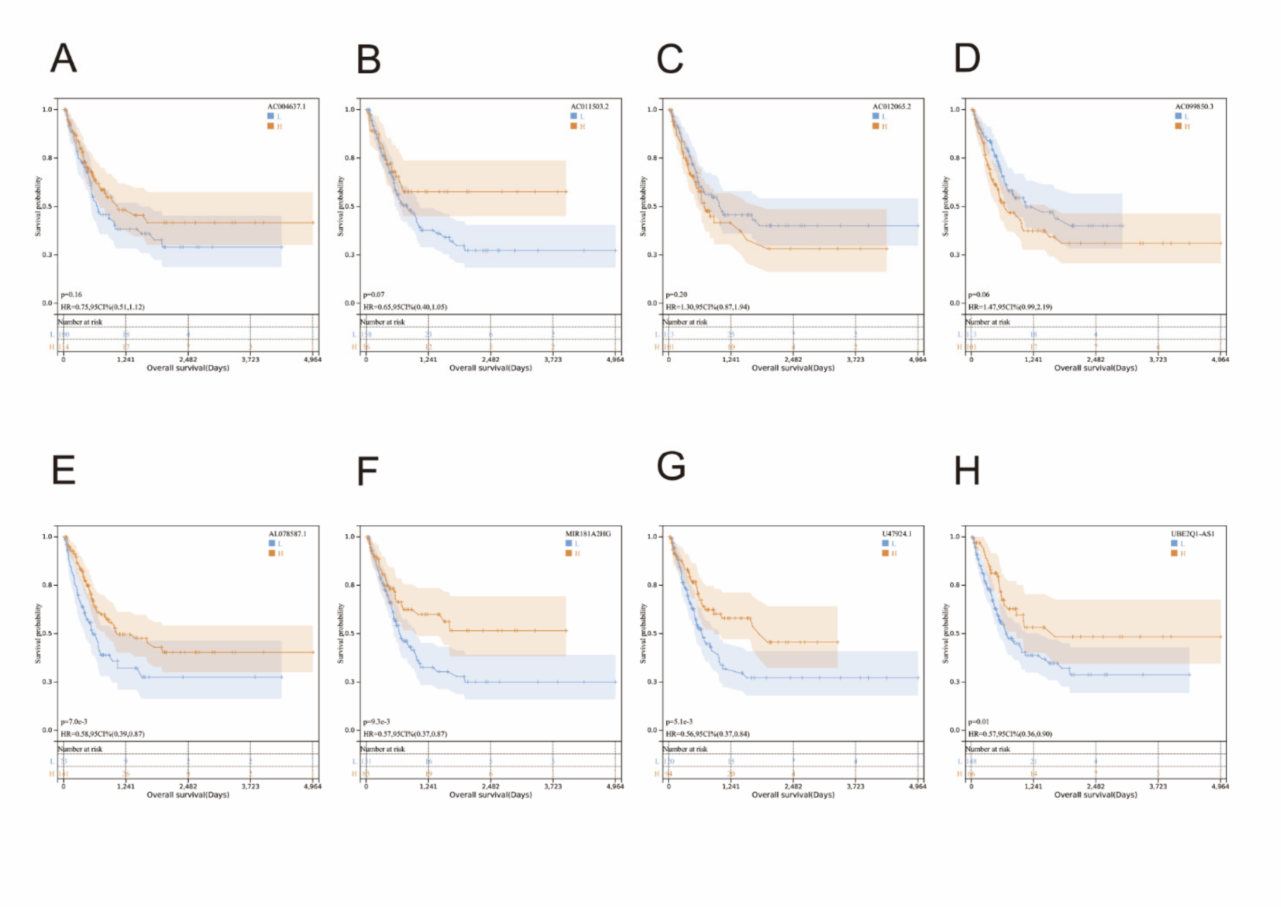

Supplementary Figure 2. A-H: Kaplan-Meier survival curves for eight genes in The Cancer Genome Atlas test set.

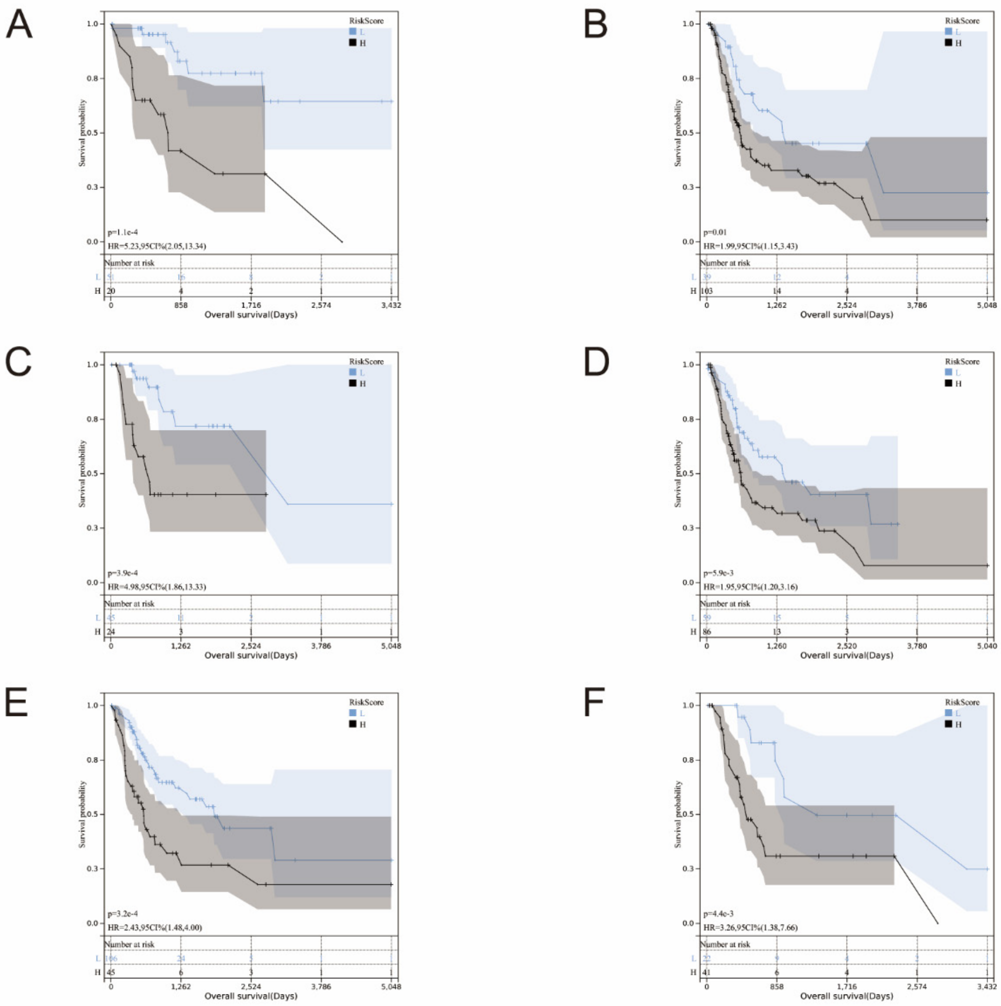

Supplementary Figure 3. A-F: All BLCA cases in The Cancer Genome Atlas training set were stratified according to clinicopathological parameters. (A) Stage I-II; (B) Stage III-IV; (C) age ≤ 60; (D) age > 60; (E) Male; (F) Female.

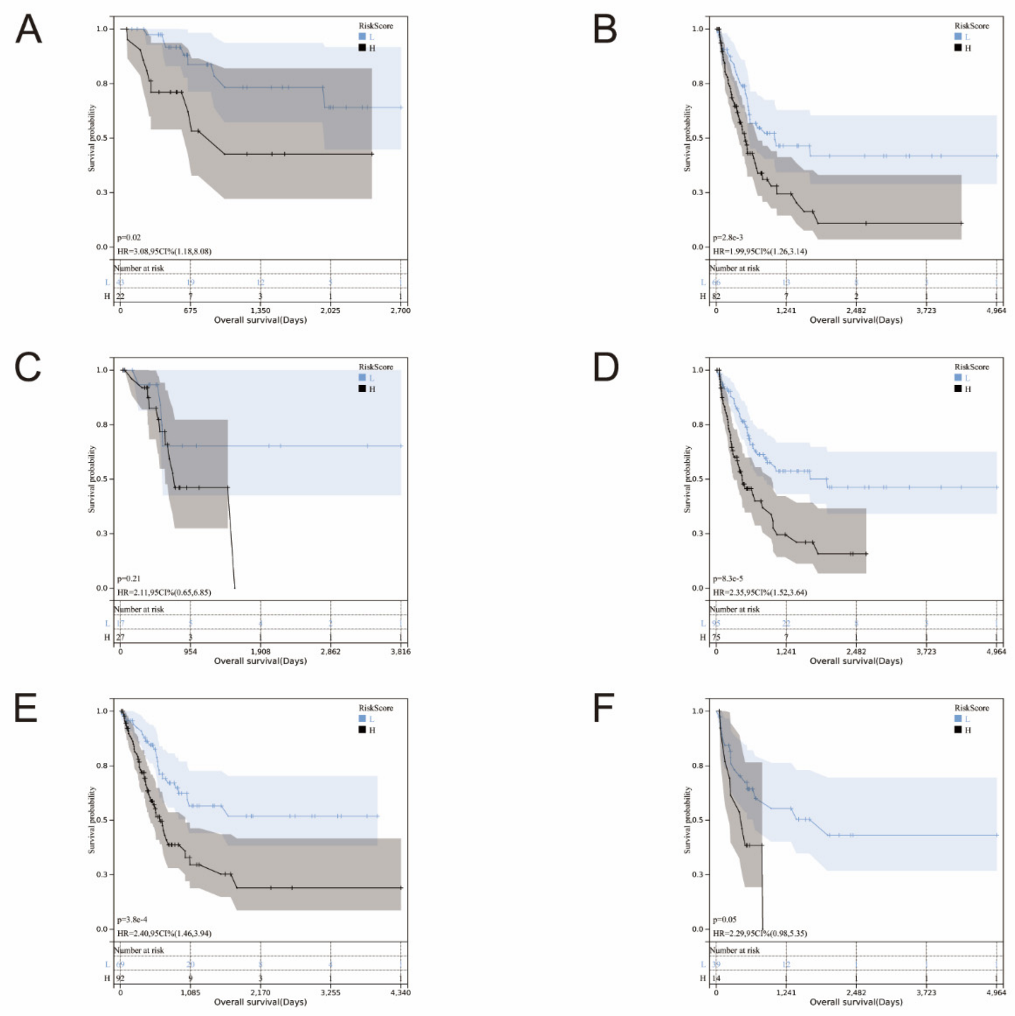

Supplementary Figure 4. A-F: All BLCA cases in The Cancer Genome Atlas test set were stratified according to clinicopathological parameters. (A) Stage I-II; (B) Stage III-IV; (C) age ≤ 60; (D) age > 60; (E) Male; (F) Female.
